# A 3D Printed Jet Mixer for Centrifugal Microfluidic Platforms

**DOI:** 10.3390/mi11070695

**Published:** 2020-07-17

**Authors:** Yunxia Wang, Yong Zhang, Zheng Qiao, Wanjun Wang

**Affiliations:** Department of Mechanical Engineering, Louisiana State University, Baton Rouge, LA 70803, USA; ywan222@lsu.edu (Y.W.); yzha148@lsu.edu (Y.Z.); zqiao1@lsu.edu (Z.Q.)

**Keywords:** three-dimensional (3D) printing, micronozzles, Y-shaped structure, mixing efficiency, histogram and standard deviation

## Abstract

Homogeneous mixing of microscopic volume fluids at low Reynolds number is of great significance for a wide range of chemical, biological, and medical applications. An efficient jet mixer with arrays of micronozzles was designed and fabricated using additive manufacturing (three-dimensional (3D) printing) technology for applications in centrifugal microfluidic platforms. The contact surface of miscible liquids was enhanced significantly by impinging plumes from two opposite arrays of micronozzles to improve mixing performance. The mixing efficiency was evaluated and compared with the commonly used Y-shaped micromixer. Effective mixing in the jet mixer was achieved within a very short timescale (3s). This 3D printed jet mixer has great potential to be implemented in applications by being incorporated into multifarious 3D printing devices in microfluidic platforms.

## 1. Introduction

Centrifugal microfluidic discs, also known as ‘‘lab-on-a-disc’’(LOD) can eliminate the requirement for an external pump and complicated fluidic interconnections. As a result, many microfluidic centrifugal systems, commonly named as lab-on-CDs, have been reported. In recent years, centrifugal microfluidic technology has found wide applications in the development of microfluidic devices for applications in biomedical fields and drug discovery [1,2,3]. They also have played a significant role in the agriculture, food engineering, spilled-oil detection and chemical industries due to the potential of becoming automated, disposable, and point-of-care (POC) clinical diagnostic tools [4,5,6,7,8]. In general, microfluidic devices have channels of scale normally less than 1mm to transport and manipulate materials, offering a portable and inexpensive possibility to operations and experiments [9]. Because only microscopic amounts of samples and reagents are consumed in such microfluidic systems, much less waste is generated.

Mixing liquids on the microscale is a challenging task because most mixing processes in microfluidic devices is limited to low Reynolds numbers caused by laminar flows in microchannels. However, for many microfluidic applications including sample dilution, reagent homogenization, chemical reactions, bioassays [10] and medical analysis [11], and materials synthesis [12], mixing is often a crucial step. An efficient mixing process helps to accelerate the rate of reaction, enhance the system sensitivity, and even affect the final product distribution. Therefore, considerable efforts have been made to develop novel micromixers to boost mixing performance in microfluidic devices for more applications in broadly various fields.

In general, micromixers can be classified into two major categories: the active and the passive ones. The active mixers always utilize an external power source and control system to assist in mixing. In general, the external power sources include vapor pneumatic power [13,14], mechanical pulsation [15], acoustic vibration [16,17], ultrasonic actuation [18], magnetic force [19,20,21,22,23], electro-kinetical force [24,25,26] and electroosmotic force [27]. The active mixers allow for more complicated processes, which always require an external instrument that adds to the size, weight, complexity and cost of the operating system. In contrast with the active types, the passive mixers generally accomplish mixing by adding geometric obstructions or altering geometries of the flow channels with no external energy sources. In terms of the mixing techniques, passive mixers can be divided into parallel flow-mixers (Y or T-types) [28,29,30,31], hydrodynamic focusing flow [32], multiple lamination flow [33,34], split and recombined flow [35], chaotic advection flow [36,37,38], structured packing flow [39], jet collision flow [40,41], recirculation flow [42], and moving droplet configuration flow-mixers [43]. Passive mixers are considered as an advantageous technique because of their simplicity and ease of fabrication in contrast with their active counterparts.

Jet collision mixing is one of the most efficient passive mixers. It maximizes the interfacial area of fluids by transforming them into plumes. The concept of impingement mixing as a microplume mixer was first investigated in some research in the late 1970s [44]. In the 1990s, the microjet mixer was comprised of micronozzle arrays to flow into a mixing chamber filled with another liquid [45]. This concept has been further optimized into two fluid streams flowing through two opposed arrays of micronozzles [41]. The fabrication of these micromixers is normally done using conventional fabrication technologies such as lithography in cleanroom facilities. In addition, bulky syringe pumps are often employed in experimental setups in those reports [41,44,45]. Three-dimensional printing, because of its fast speed and low cost, and wide choice of materials, has become an attractive tool for fabrication for micro-meso fluidics and mechanical systems. In addition, 3D printed models can be directly applied into testing and even as end products.

The fast-developing additive manufacturing (AM) technology with reasonable fabrication efficiency has provided a powerful tool to fabricate high precision, integrated microfluidic systems. Three-dimensional-printed microfluidics is suitable for a wide range of applications such as immunoassays [46], material science [47], particle analysis [48] and extraction of preterm birth biomarkers [49]. In addition to many other applications, much research in applying 3D printing technologies to make mixers have also been reported in recent years for different applications. The 3D printed mixer with integrated staggered herringbones has been studied to exploit the limits of 3D flow magnetic resonance imaging (MRI) for the analysis of fluid dynamics on a sub-millimeter scale [50]. Using high-definition MultiJet 3D printing to remake five different passive micromixers has also been reported [51]. The indirect 3D printing method was used to fabricate a helical and a cubic electrode mixer and to increase the mass transfer up to 47% the standard flat electrode by some researchers [52]. The 3D printed modular mixing components operate on the basis of splitting and recombining fluid streams to decrease interstream diffusion length [53]. A 3-inlet 3D printed, disposable, high-throughput micromixer for production of therapeutic nanoparticles with syringe pumps has also been studied [54].

One of the commonly used additive manufacturing (AM) technologies, fused deposition modelling (FDM) has emerged as a rapid prototyping approach for various applications in recent years. FDM was first reported to fabricate templates for soft lithography of microfluidic devices with PDMS in 2002 [55]. FDM was demonstrated as the direct fabrication method for microfluidic chemical reaction-ware in 2012 [56]. Compared with other 3D printing technologies such as stereolithography (SLA) and manufacturing using femtosecond laser, FDM is much cheaper and easier to use, and faster [57]. However, it has the drawback of lower resolution, and therefore larger minimum feature sizes in final products.

In this paper, we report a jet collision micromixer (jet mixer) fabricated using a low-cost FDM printer. The micromixer is designed to have two opposed arrays of micronozzle to generate microplumes to enhance the mixing efficiency by both quick diffusion and turbulent mechanisms. By controlling the distance between the neighboring filament lines and also taking on the additive manufacturing nature of FDM, a jet mixer with large arrays of micronozzles (microchannels) was easily generated in a vertical direction. The mixer was installed on a centrifugal fluidic platform for testing. Flow visualization by high-speed camera demonstrated that the mixing process was complete within a few seconds. The mixing efficiency of the jet mixer was experimentally measured by contrastively analyzing with a Y-shaped mixer.

## 2. Principle and Design of the Jet Mixer

**Figure 1:**
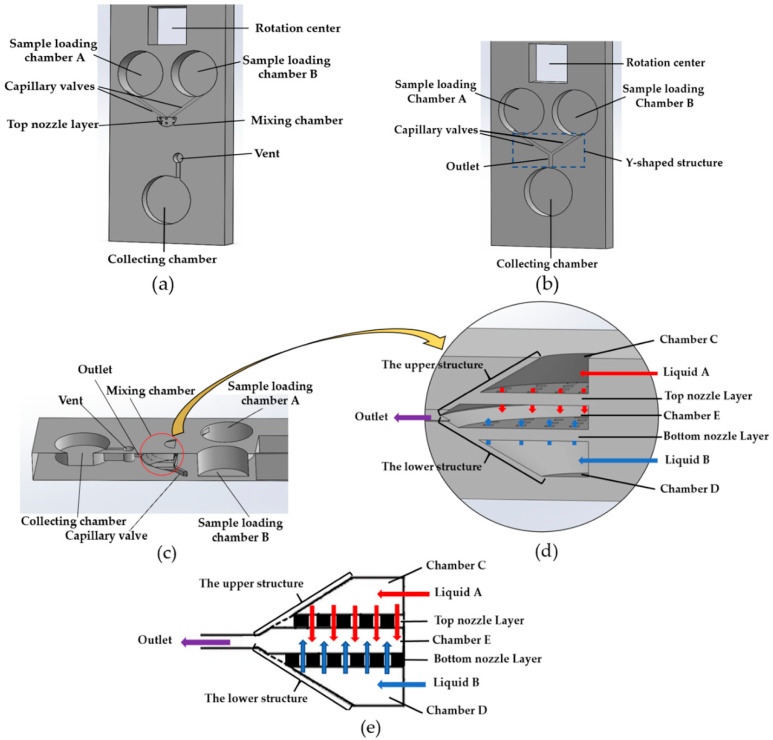
Design of the 3D jet mixer: (**a**) the exploded view of the assembly of the jet mixer to show the components; (**b**) design of the Y-shaped micromixer for comparison with the jet mixer’s performance; (**c**) the cross-sectional view on the right plane; (**d**) detailed design of the mixing chamber; (**e**) schematic diagram of the mixing chamber on the right plane.

The design of the jet mixer and Y-shaped mixer in this paper is schematically demonstrated in Figure 1. Figure 1a–d were created via using Solidworks (Dassault Systems SolidWorks Corp, Waltham, MA, USA). Figure 1a shows the general layout of the mixer with the sample loading chambers A and B (loading liquid volume 100 μL for each chamber), the mixing chamber, the vent, and the collecting chamber (200 μL). The capillary valves connecting the sample loading chamber and the mixing chamber were designed to have a cross-section of 0.8 mm (width) × 0.5 mm (height). The corresponding cross-sectional view of the jet mixer is shown in Figure 1c. At the beginning of the mixer operation, the sample liquids A and B were preloaded into the sample loading chambers A and B separately. The mixer was then installed on the platform to rotate. When the rotational speed of the platform reached the burst frequency, the capillary valves were turned to open, centrifugal forces helped to propel the liquid samples A and B to flow into the mixing chamber. Figure 1d and e show the more detailed schematic.

The design of the mixing chamber: the mixing chamber contains two opposite arrays of micronozzles, which divide this chamber into three subchambers C, D and E. During the mixing process, two different liquid samples (A and B) are propelled into the chambers C and D, respectively, and then flow into chamber E to mix through the two up-down arrays of micronozzles (Figure 1e). Finally, the mixed liquid is delivered to the collecting chamber. The vents are designed for air release.

In fabricating the two arrays of micronozzles, the smaller filament size was created from an extruder primarily by increasing the distance between neighboring filament lines to form micronozzles. In the additive manufacturing process with FDM, the structure was made in a layer by layer fashion. As a result, the micronozzles can be “naturally” generated in a vertical direction. This is the main reason why the entire layout for the mixing chamber is in a vertical direction instead of in the horizontal plane.

For the sake of comparison, a Y-shaped mixer was also designed, built, and tested. The design of a Y-shaped mixer can be fully realized by a 3D FDM printer, to achieve optimal mixing performance. The design of the Y-shaped mixer is schematically shown in Figure 1b. It consists of sample loading chambers A and B, a collecting chamber, and a Y-shaped structure of channels with capillary valves and an outlet. All the chambers of the Y-shaped mixer are in the same plane, which is different from the placement of the chambers in the jet mixer. In the jet mixer, jet collision was designed to happen in a vertical (up-down) direction, not in a right-left direction. This was due to the limitation of FDM printer that can be used to produce arrays of nozzles in a vertical direction. Because chambers C and D are designed to face each other in a vertical direction and are also connected with chambers A and B, respectively, this design limitation requires an increased depth for chambers A and B. To have the same designed volumes of chambers A and B for both the Y-shaped mixer and the jet mixer, the loading chambers A and B for the jet mixer and Y-shaped mixer are designed to have the same volume but different diameters and depths as marked in Figure 1.

The theoretical burst frequencies of the capillary valves for both the Y-shaped mixer and the jet mixer are designed to be the same. The burst frequency $f_{b}$ of a capillary valve is defined in terms of rotations per minute (RPM), and can be calculated using the following equation:

(1) $$f_{b}=\sqrt{\frac{P_{cap}}{\rho \Delta R\bar{R}}}(\frac{30}{\pi })\;$$

where, $P_{cap}$ = $4cos\theta \Upsilon /D_{h}$, *ρ* is the density of the liquid, Δ*R* is the difference between the top and bottom of the liquid levels with respect to the rotation center, $\bar{R}$ is the average distance of the liquid to the rotation center,$\;P_{cap}$ is the capillary pressure, Υ is the surface tension, *θ* is the contact angle, and $D_{h\;}$ is the hydraulic diameter. With the design dimensions shown in Table 1, the theoretical burst frequency was estimated to be 473.31 RPM.

The main difference between the jet mixer and Y-shaped mixer is the extra mixing chamber in the jet mixer. In the Y-shaped mixer, the fluid samples from loading Chambers A and B flow through the Y-shaped channels and enter the collecting chamber directly. The collection chamber also serves as the mixing chamber in the Y-shaped mixer. In comparison, in the design of the jet mixer, the fluid samples from the two loading chambers must flow through two arrays of micronozzles in the mixing chamber before it enters the collecting chamber in which additional mixing happens. The dimensions of the capillary valves (the flow channels to the mixing chambers) for both types of mixers are designed to be the same. The nominal flow resistances are therefore designed to be the same. By assuming the flows in these microchannels are laminar, the nominal flow resistances are calculated to be 1.76 × 10^9^ (Pa·s/m^3^).

**Figure 2:**
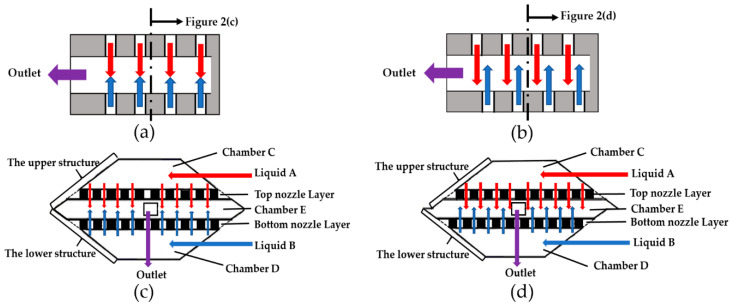
Design options of the jet-impinge mixer: (**a**) Opposite micronozzles directly facing each other; (**b**) Opposite side micronozzles, off-set; (**c**) Cross-section on the top plane of design (**a**); (**d**) Cross-section on the top plane of design (**b**).

Figure 2 shows common design options for the arrays of the micronozzles. There are normally two options to arrange the two facing arrays of impinging jets: directly facing as shown in Figure 2a, or to have a fixed shift as shown in Figure 2b. Figure 2a,b are common design principles for jet flow collision, we designed our mixing chamber based on these principles. The cross-section diagram of designs (a) and (b) is shown graphically in Figure 2c,d, respectively. The function of two facing arrays of micronozzles is to transform liquid samples into microjets. The liquid samples are driven by centrifugal force to feed through multiple micronozzles, converting liquids into impinging plumes and mixing in Chamber E.

As shown in Figure 2c,d, the sample liquids (A and B) are simultaneously introduced into the Chambers C and D in up and down directions in our design. After introduction, the location of the sample liquids become upper and lower plane instead of the same plane. These micro-plumes of flows are introduced oppositely into chamber E, impinging upon each other, either face-to-face as shown in Figure 2c or passing by each other as shown in Figure 2d. The design of Figure 2d obviously has the advantage of maximizing the effective contact surfaces between the impinging plumes, and therefore boosting the diffusion process. The off-set also makes it more likely to produce more vortexes due to flowing across the mixing chamber from one side to the other [41]. It was therefore the preferred design for the mixer.

## 3. Fabrication of the Jet Mixer

The jet mixer was 3D printed using an FDM 3D printer (Ultimaker 3). Simplify3D software was used to adjust the relevant parameters such as nozzle diameter, extrusion width, primary layer height and layer modifications in achieving the key feature (the micronozzles) and the designed dimension of the jet mixer. The following operational parameters were used in the fabrication process: (1) layer thickness for top and bottom nozzle layer, 0.05 mm; (2) layer thickness for other components, 0.15 mm; (3) nozzle diameter, 400 μm; (4) flow rate, 0.9; (5) print speed, 3600 mm/min; (6) nozzle temperature, 215 °C; (7) heating bed temperature, 55 °C; (8) spacing between neighboring filaments, 600 μm; (9) heated building chamber (25 °C).

**Figure 3:**
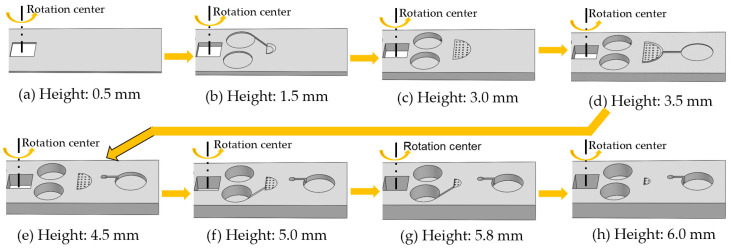
The flowchart of the additive manufacturing process for the jet mixer cartridge: (**a**) the substrate layer (height: 0.0–1.0 mm); (**b**) the mixing Chamber D layer (height: 1.0–2.5 mm); (**c**) the bottom nozzle layer (height: 2.5–3 mm); (**d**) the mixing Chamber E layer (height: 3.0–4.0 mm); (**e**) the top nozzle layer (height: 4.0–4.5 mm); (**f**) the mixing Chamber C layer (height: 4.5–6.0 mm); (**g**) the final geometry of the jet mixer before the cover was made; (**h**) the completed jet mixer. (Process 1-1: 0.0–2.5 mm; Process 2-2: 2.5–3.0 mm; Process 3-3: 3.0–4.0 mm; Process 4-4: 4.0–4.5 mm; Process 5-5: 4.5–6 mm).

White colored PLA (Polylactic Acid, from Dynamism, Chicago, IL, USA) was used as the structural material (and therefore the printer filament) for the jet mixer. We designed the whole model using Solidworks (Dassault Systems SolidWorks Corp, Waltham, MA, USA). Figure 3 shows the 3D printing process of the jet mixer layer by layer with FDM. It took about one and a half hours to print one prototype jet mixer with the overall size of 100 mm (in length) × 24 mm (in width) × 6 mm (in height). The entire fabrication process was divided into 5 processes in Simplify3D software: Process 1-1, Process 2-2, Process 3-3, Process 4-4 and Process 5-5 in Figure 3. As shown in Figure 3a,b, in Process 1-1, the structure’s height increased from 0 mm to 2.5 mm. It consists of the substrate layer (0–1 mm) and chamber D (2.00 mm (lower radius), 5.46 mm (upper radius), 2.00 mm (H)) in the mixing chamber, the sample loading chambers A and B, and the connection capillary valve between sample loading Chamber B and the Chamber B of the mixing chamber. The height of the bottom nozzle layer in the mixing chamber was set to be 0.5 mm, corresponding to the Process 2-2 from 2.5 mm to 3 mm in Figure 3c. In Figure 3d, the Chamber E (6.33 mm (lower radius), 7.20 mm (upper radius), 2 × 0.50 mm (H)) was formed as a hollow structure in the mixing chamber with its height increased from 3 mm to 4 mm as shown in the Process 3-3. Meanwhile, the deposition for the output channel and collecting chamber for collecting mixed fluid were started. Figure 3e shows the top nozzle layer starting to be printed on the hollow Chamber E from 4 mm to 4.5 mm in the Process 4-4. The Process 5-5 is shown in Figure 3f–h, the height of the printed structure increased from 4.5 mm to 6 mm. The fabrication of Chamber C (2.00 mm (lower radius), 5.46 mm (upper radius), 2.00 mm (H)) of the mixing chamber, and the other capillary valve connecting sample loading Chamber A to Chamber C of the mixing chamber started at height 5.0 mm in Figure 3f. Figure 3g depicts the structure of the jet mixer before the cover was made. Figure 3h is the finished 3D FDM-printed jet mixer.

**Figure 4:**
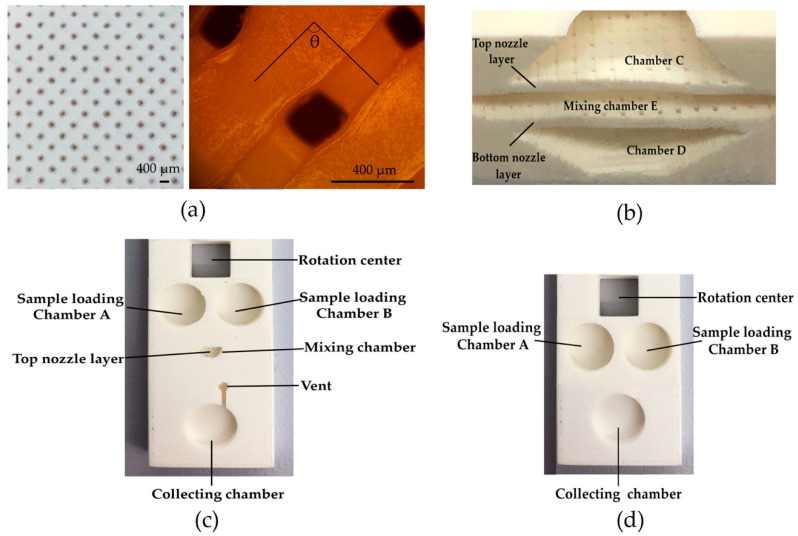
(**a**) A photo image of the 3D printed micronozzles; (**b**) a microscope image of the 3D printed micronozzles, θ = 90°; (**c**) a photo image of the 3D printed jet mixer; (**d**) a photo image of the 3D printed Y-shaped mixer.

The photo images of the 3D printed micronozzles and jet mixer with PLA as structural material are shown in Figure 4, Figure 4a is the photo image of the micronozzles. Figure 4b shows the image of the micronozzles as captured by LED Digital Binocular Compound Microscope (AmScope, Orange County, CA, USA). In 3D software, rectilinear infill patterns with infill angles in +45° and −45° directions were chosen for automatically forming micronozzles during the 3D printing process. The angle formed by two adjacent print layers is therefore 90° as shown in Figure 4b. It can be observed that the largest size of micronozzle was 200 μm by 250 μm. The smallest size was 50 μm by 50 μm. This wide range of size variation was caused by the error of the low-cost FDM printer used to fabricate the mixer. The size of the micronozzles can be adjusted according to design requirements by changing the settings of the extruder, the thickness of each printing layer, the infill, as well as other printer parameters in Simplify3D software. Two different types of sandpaper were then used to polish the 3D printed micromixer. The fabrication of the jet mixer took about one and a half hours to complete. It was time-saving, labor-saving, cost-effective and easy-to-make in comparison with other technologies such as lithography. Finally, transparent polypropylene adhesive tapes (Scotch tape, 3M) were applied to seal the micromixer. The vent holes were punched on the covering tape to prevent air pressure building up. The finished 3D printed jet mixer is shown in Figure 4c. Figure 4d shows a photo image of the 3D printed Y-shaped mixer.

In our design of the jet mixer, two arrays of impinging micronozzles were arranged in the vertical or out of plane orientation instead of horizontal or in plane. The main reason for choosing such a design layout is because of the resolution limitation of the 3D printer used in the fabrication. It is difficult to make arrays of micronozzles in plane with the desired resolution, depending on purpose-designed micronozzles in Solidworks. The unique advantage of the fused filament fabrication (FFF) in the FDM printer is that it makes it much easier to form arrays of micronozzles automatically in a vertical direction by manipulating the filament size from the extruder in selecting the infill pattern in the Simplify3D software. It should be noted the line spacing in the FDM printer is normally considered a drawback because it lowers the surface quality of the finished structure. For the fabrication of the jet mixer reported in this paper, it actually helped to simplify the fabrication process.

## 4. Experimental Results and Discussions

### 4.1. The Experimental Setup

**Figure 5:**
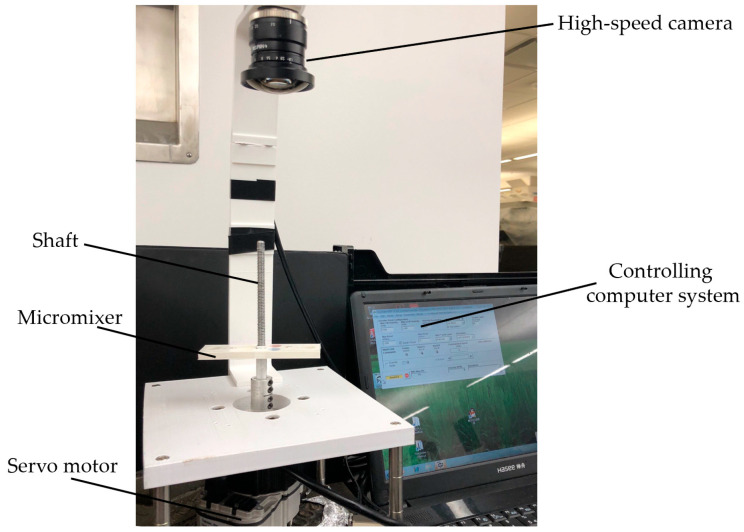
The experimental setup of the centrifugal platform with 3D microfluidic cartridge mounted.

Figure 5 shows a photo image of the centrifugal platform setup to test the micromixers. The micromixer was mounted on the DC servo motor controlled by a laptop computer. A high-speed camera with frame rate 80 fps (Pixelink, Rochester, NY, USA) was installed above the fluidic system to monitor the mixing processing in real time. A series of experiments were conducted to test the mixing performances of the jet mixer. Experiments with a simple Y-shaped mixer which is one of the most commonly used and extensively studied, were also conducted for comparison.

### 4.2. Mixing Efficiencies and Discussions

In the experiments, 100 μL of red and blue dyed deionized (DI) water were injected into loading Chambers A and B first. Experiments were first run using blue dyed DI water and red dyed DI water because it is easy to observe under a high-speed camera. The mixers were then attached to the rotational platform for tests. The mixers were installed in an orientation so that the loading chambers were located closer to the shaft and the collecting chambers were toward the edge of the platform along the radial direction. The centrifugal platform was driven to rotate in a counter-clockwise direction.

**Figure 6:**
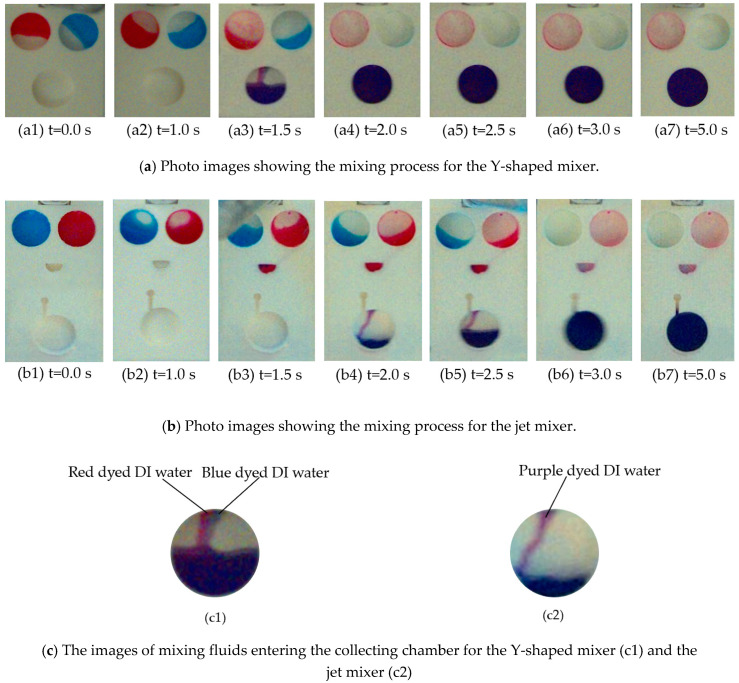
Photo images showing the experimental results: (**a**) for the Y-shaped mixer; (**b**) for the jet mixer; (**c**) the images of fluid entering collecting chambers: (**c1**) for the Y-shaped mixer chambers and (**c2**) for the jet mixer. The rotation of the platform was brought to a complete stop at t = 5s.

It should be noted that the burst frequencies for the capillary valves in both the Y-shaped and the jet mixer were designed to be the same at 473.37 RPM. However, because of fabrication errors in the microchannels, the burst frequencies were found to be much higher. When the rotational speed of the micromixer was increased to 1000 RPM, the capillary valves were turned open roughly at the same time for both the Y-shaped mixer and the micro-jet one. The red and blue dye samples in the loading chambers A and B were released and flowed through the Y-shaped channels and entered the collecting chamber as shown by the sequential photo images in Figure 6(a1–a7). At the same time, the mixing process for the jet mixer is shown by the sequential photo images of Figure 6(b1–b7). The blue and red dyes flowed into the two facing arrays of micronozzles, and then entered the mixing chamber, and were finally released into the collecting chamber. The mixing process was recorded in real time by the monitoring camera at the rate of two pictures per second.

**Figure 7:**
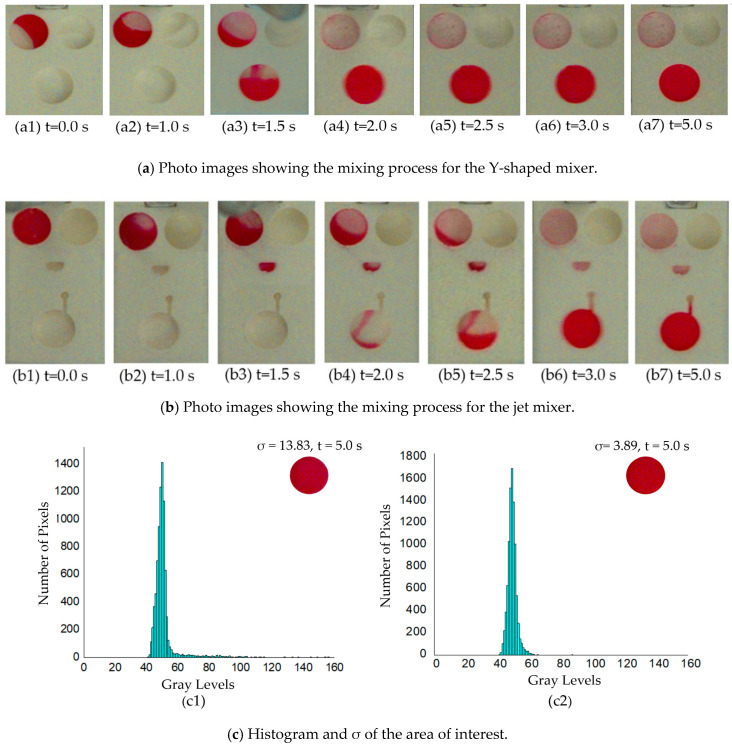
Analysis of the mixing performances for the two types of mixers. Photo images showing the experimental results for (**a**) the Y-shaped and (**b**) the jet mixer; (**c**) histogram and σ of the photo image of the mixed fluid samples for (**c1**) the Y-shaped mixer and (**c2**) the jet mixer.

At the beginning of the experiments, the two loading chambers were filled with blue and red solutions as shown in Figure 6(a1,b1). In the experiments, the same volumes of the sample fluids, 100 μl, were preloaded into chambers A and B. However, because chambers A and B for the jet mixer have a slightly smaller diameter and larger depth than those of the Y-shaped mixer, the sample fluids completely covered the bottom surfaces of both chambers in the jet mixer and only partially covered the loading chambers for the Y-shaped mixer. This made the photo image Figure 6(a1) seem to complete the mixing for the Y-shaped mixer. For the jet mixer, the mixing time was a little longer, because the flow resistance of the two arrays of micronozzles slowed the flow rate of the fluid samples. As can be observed from Figure 6(b1–b7), it took more than 0.5 s before the blue and red dyes entered the collecting chambers through the arrays of micronozzles. It took about 3 s for the two loading chambers with blue and red dyes to be emptied and the mixed solution to flow into the collecting chamber for the jet mixer. When the mixing was completed after 5 s, the rotation of the platform was stopped. The photo images in Figure 6(a7,b7) were taken in the resting condition of the platform to improve image resolution for more accurate analysis of the mixing effects for both mixers as shown in Figure 7(c1,c2). It should be noted that the collecting chamber in Figure 6(b6) was already empty: because the micronozzles were stained by the dye in the fluids, the photo image looks like there was still some fluid leftover. Figure 6(c1,c2) show the situation of the mixing fluids flowing into the collection chambers for the two types of mixers. The red and blue dyed DI water flowed in parallel into the collecting chamber of the Y-shaped mixer at 1.5 s (Figure 6(c1) corresponding to Figure 6(a3)), indicating that the mixing mainly happened in the collecting chamber due to the vortex. As displayed in Figure 6(c2) corresponding to Figure 6(b4), purple dyed DI water was propelled into the collection chamber of the jet mixer at 2 s, this meant that the red and blue dyed DI water had already been mixed well in the mixing chamber. Injecting into the collection chamber helps to further mix the sample fluid. The time difference for the mixing to complete in Figure 6 is mainly due to the fact that mixing fluids flow through different structures in the Y-shaped mixer and the jet mixer to enter the collecting chambers. Flow resistance from the two arrays of micronozzles in the mixing chamber of the jet mixer slowed the flow rate of the fluid samples.

Standard deviation (σ) is a common method in analyzing mixing performance. Greyscale distribution for the colorless DI water and the red dyed DI water are about from 200–255 and 0–50, respectively. Therefore, experiments were also run using colorless DI water and red dyed DI water to check the mixing efficiencies. The whole experimental process was shown in Figure 7a,b. The mixing process of colorless and red dyed DI water under the observation of a high-speed camera was the same as that obtained using red and blue dyed DI water in Figure 6a,b. The images of the final mixed solution in the collecting chamber were processed and analyzed by using the software «Image J» (NIH Image, Bethesda, MD, USA) and «Origin 8.5» (OriginLab, Northampton, MA, USA) to obtain the σ and the distribution of the gray intensity histogram. The histogram and the corresponding σ for both micromixers are shown in Figure 7(c1,c2).

To quantify the effectiveness of the two different types of mixers, the standard deviations σ of the pixel intensity of the images of the mixed solutions in the collecting chambers were utilized to numerically represent the mixing effectiveness. The standard deviation σ, defined by the square root of the variance, was used to measure the dispersion of the data values. The data points which are near the mean value in the histogram with low values of σ indicate higher mixing effect. Oppositely, the data points that have widely spread (with the high σ), indicate lower mixing effect. For the final mixed solutions shown in images in Figure 7(a7,b7), the areas of interest were the region of the collecting chambers, which were digitally cut out for analysis. Each of the images of the collecting chambers was analyzed by employing the software «Image J» and «Origin» for further processing to get a histogram plot, the frequency of the pixel’s gray levels of the regions was plotted. The gray intensities of all the pixels were obtained and the σ of each image was obtained. The x-axis of the histogram graph represents the gray levels and the y-axis represents the number of pixels in that specific tone. Figure 7(c1) shows the distribution of pixels for the mixed fluid sample in the collecting chamber for the jet mixer was less dispersed and more concentrated (gray levels from 40 to 60) in contrast with that for the Y-shaped mixers as shown in Figure 7(c2) (gray levels from 40 to 160). Therefore, the histogram distribution displays the jet mixer has a much better mixing efficiency. The change in σ of the mixed liquids for the two mixers is shown in Figure 7(c1,c2). Based on the foregoing analysis, the smaller σ is, the better the mixing efficiency. The σ for both images shown in Figure 7(c1,c2) was 13.83 for the Y-shaped mixer and 3.89 for the jet mixer. Hence, the mixing efficiency of the jet mixer can be considered as 3.56 times of that for the Y-shaped one. Very obviously, the two facing arrays of micronozzles have helped to enhance the mixing efficiency dramatically. The mixing performance of the jet mixer outperformed that of the Y-shaped mixer.

Finally, the experiments were repeated five times for both mixers. The corresponding results are shown in Table 2. The average σ of the jet mixer was found to be 3.56 (3.89, 3.59, 3.24, 3.89, 3.21), in comparison with the average σ of 13.77 for the Y-shaped mixer in five tests (13.53, 13.83, 13.47, 13.88, 14.15). Furthermore, the σ for the five standard deviations of both mixers was 0.25 (Y-shaped mixer) and 0.29 (jet mixer). In addition, the required mixing time for colorless DI water and red dye colored DI water of the jet mixer is shorter than that reported by Esmail et al. [58] using the moment of inertia mixing method in the second mixing cycle. It should be noted that using the image analysis method to estimate mixing performance is limited by the high-speed camera resolution and the irregular shape of the liquid samples in the collecting chamber. However, the σ of the gray intensities can still be a useful estimation for mixing effectiveness. In addition, in the design of traditional Y-shaped mixers, S-shaped flow channels are used to further improve the mixing efficiency. For both the Y-shaped mixer and the jet mixer in this paper, S-shaped channels could also be added for better mixing efficiencies.

## 5. Conclusions

A jet mixer with two facing arrays of micronozzles was designed and successfully fabricated using 3D printing technology. The micronozzles were realized by using Simplify3D software to change the size of filament and easily printed using a low-cost FDM printer. Experiments were conducted to study the mixing efficiencies of the jet mixer and compared with the commonly used Y-shaped mixer. The jet mixer has demonstrated very good mixing efficiency with an average σ of 3.56 for colorless DI water and red dye colored DI water with a mixing time of less than 5 s. The main reason behind the dramatically enhanced mixing efficiency of the jet mixer is the mixing chamber that includes two facing arrays of micronozzles. This unique fabrication method makes it possible to print micro-nozzles via an increased distance between neighboring filament lines. The design significantly increased the effective contact areas of the two liquid samples by impinging plumes from two opposite arrays of micronozzles and significantly enhanced the mixing efficiency. The study also proved that FDM 3D printing technology can be used in the fabrication of the jet mixer and is time-saving, labor-saving and cost-effective. PLA was used as the structural material for demonstration purposes only. Many other materials can be used in FDM and other 3D printing technologies for specific applications and needs. Additionally, 3D printing technology made it very easy to fabricate complete microfluidic CDs without extra equipment installation. It may help to simplify the design, reduce the cost of production, and enable the development of novel fluidic systems for many applications.

## Figures and Tables

**Table 1:** The dimensions of the common components for the jet mixer and Y-shaped mixer (radius—R, height—H, width—W).

Components	Dimensions in Jet-Mixer	Dimensions In Y-Shaped Mixer
Sample Loading Chamber A	3.37 mm (R) × 5.00 mm (H)	4.77 mm (R) × 2.50 mm (H)
Sample Loading Chamber B	3.37 mm (R) × 5.00 mm (H)	4.77 mm (R) × 2.50 mm (H)
Capillary Valve	0.80 mm (W) × 0.50 mm (H)	0.80 mm (W) × 0.50 mm (H)
Outlet	0.80 mm (W) × 0.50 mm (H)	0.80 mm (W) × 0.50 mm (H)
Collecting Chamber	4.81 mm (R) × 2.75mm (H)	5.05 mm (R) × 2.50 mm (H)

**Table 2:** The standard deviation value of the Y-shaped and the jet mixer under different numbers of the test.

Test #	Standard Deviation of Jet-Mixer	Standard Deviation of Y-Shaped Mixer
1	3.89	13.53
2	3.59	13.83
3	3.24	13.47
4	3.89	13.88
5	3.21	14.15
